# Favorable Prognosis in Patients with Recovered Pulmonary Hypertension after TAVI: An Analysis of the LAPLACE-TAVI Registry

**DOI:** 10.3390/jcm12020729

**Published:** 2023-01-16

**Authors:** Takuma Koike, Hiroshi Iwata, Yuichi Chikata, Shinichiro Doi, Ryo Naito, Hidetoshi Yasuda, Takehiro Funamizu, Hirohisa Endo, Sakiko Miyazaki, Shinya Okazaki, Ryosuke Higuchi, Itaru Takamisawa, Kei Sato, Harutoshi Tamura, Hiroaki Yokoyama, Tetsuya Tobaru, Shuichiro Takanashi, Minoru Tabata, Tohru Minamino

**Affiliations:** 1 Department of Cardiovascular Biology and Medicine, Juntendo University Graduate School of Medicine, Tokyo 113-8421, Japan; 2 Department of Cardiology, Sakakibara Heart Institute, Tokyo 183-0003, Japan; 3 Department of Cardiology and Nephrology, Mie University Graduate School of Medicine, Mie 514-8507, Japan; 4 Department of Cardiology, Pulmonology and Nephrology, Yamagata University School of Medicine, Yamagata 990-9585, Japan; 5 Department of Cardiology and Nephrology, Hirosaki University Graduate School of Medicine, Aomori 036-8652, Japan; 6 Department of Cardiology, Kawasaki Saiwai Hospital, Kanagawa 212-0014, Japan; 7 Department of Cardiovascular Surgery, Kawasaki Saiwai Hospital, Kanagawa 212-0014, Japan; 8 Department of Cardiovascular Surgery, Sakakibara Heart Institute, Tokyo 183-0003, Japan; 9 Department of Cardiovascular Surgery, Juntendo University Graduate School of Medicine, Tokyo 113-8421, Japan

**Keywords:** TAVI, prognosis, TRPG, pulmonary hypertension, echocardiography, severe AS

## Abstract

Pulmonary hypertension (PH) is a common complication of aortic stenosis (AS). Despite the established association between PH and poor outcomes in patients with AS, the prognostic implication of a change in PH after transcatheter aortic valve implantation (TAVI) has been rarely evaluated. This study analyzed a prospective multi-center TAVI registry database involving six Japanese centers and used the transtricuspid pressure gradient (TRPG) obtained by echocardiography to estimate pulmonary artery systolic pressure. The participants (*n* = 2056) were first divided into two groups by TRPG before TAVI, a PH (−) group (TRPG < 30 mmHg) (*n* = 1407, 61.9%) and a PH (+) group (TRPG ≥ 30 mmHg) (*n* = 649, 28.6%). Next, by TRPG after (4.1 ± 5.3 days) TAVI, the PH (+) group was further subdivided into two groups, Recovered PH (TRPG < 30 mmHg, *n* = 253) and Persistent PH (TRPG after TAVI ≥ 30 mmHg, *n* = 396). The median follow-up duration was 1.8 years. The primary and secondary endpoints were the composite and each of cardiovascular (CV) death and heart failure hospitalization, respectively. Unadjusted Kaplan-Meier estimates with log-rank comparisons showed significantly higher cumulative incidences of primary and secondary endpoints in the Persistent PH group compared to other groups. Moreover, adjusted multivariate Cox-proportional hazard analyses showed that a decreased (−10 mmHg) TRPG after TAVI was linearly associated with a reduced risk of the primary endpoint (hazard ratio (HR): 0.76, 95% confidence interval (CI): 0.64–0.90, *p* = 0.0020). The findings in the present study indicate that the recovery of PH may partly contributes to the prognostic benefit of TAVI procedure in patients with AS and elevated pulmonary artery systolic pressure.

## 1. Introduction

Aortic valve stenosis (AS) is currently the most common valvular heart disease and its prevalence is increasing due to aging of the population worldwide [1]. Although surgical aortic valve replacement (SAVR) had been the only curative therapeutic option for patients with severe AS for a long time, transcatheter aortic valve implantation (TAVI) has rapidly expanded its indication since its introduction, although it was originally used in patients at high-risk or those deemed inoperable for SAVR [2,3]. However, the advancement of transcatheter valve systems, increased operator experience, and technical refinements in the procedures have led to an expansion of the TAVI indication for patients who are at lower (low or intermediate) risk [3,4,5,6]. Therefore, in accordance with the increased number of patients assessed for TAVI, more accurate preoperative risk assessment and prediction of longer-term outcomes is needed [7,8].

In AS patients, PH is frequently complicated [9], as increased afterload pathologically induces an elevation in left atrial pressure which increases pulmonary arteriolar tone followed by reactive PH [10]. Previous studies involving patients undergoing SAVR showed a significantly elevated risk of postoperative mortality in AS patients with PH before surgery [11]. Moreover, a reduction in systolic pulmonary arterial pressure by SAVR in patients with AS and PH was shown to be associated with better outcomes [12,13,14]. However, in AS patients undergoing TAVI, data regarding the prognostic impact of recovery of PH after TAVI on long-term outcomes are limited. Therefore, this retrospective cohort analysis of a prospective multicenter TAVI registry database consisting of six Japanese centers aimed to address this gap in the literature through assessing the impact of change in PH after TAVI, compared to baseline on long-term outcomes, using non-invasive echocardiographic measurement of TRPG to estimate pulmonary artery pressure [15].

## 2. Materials and Methods

### 2.1. Study Population

This study is a retrospective analysis of a prospective multi-center registry database of patients who underwent TAVI at six centers (two municipal and four university hospitals) in Japan, termed The aLliAnce for exPloring cLinical prospects of AortiC valvE disease (LAPLACE TAVI registry). The participating hospitals were the Sakakibara Heart Institute, Juntendo University Hospital, Yamagata University Hospital, Hirosaki University Hospital, Mie University Hospital, and Kawasaki Saiwai Hospital. This study was performed in accordance with the Declaration of Helsinki and with approval from the Institutional Review Board (IRB) of Sakakibara Heart Institute (IRB-ID: 17-048), Juntendo University Hospital (IRB-ID: 17-263), Yamagata University Hospital (IRB-ID: 2019-407), Hirosaki University Hospital (IRB-ID: 2020-040), Mie University Hospital (IRB-ID: H2021-049), and Kawasaki Saiwai Hospital (IRB-ID: R4-13), respectively, and the registry is publicly registered in the University Medical Information Network Japan-Clinical Trials Registry, (UMIN000031133). Written informed consent was obtained from all participants for this registry.

### 2.2. TRPG, E/A and E/e’ Estimation by Pre- and Post TAVI Echocardiography

All participants underwent two-dimensional and Doppler transthoracic echocardiography (TTE) at each center according to the guidelines [16]. The timing of pre-TAVI TTE was 33.3 ± 33.8 days prior and that of post-TAVI was 4.1 ± 5.3 days after the TAVI procedure, respectively. Tricuspid regurgitation (TR) was evaluated either in the apical 4-chamber, parasternal short-axis, or right ventricular inflow view. Grades of TR and mitral regurgitation (MR) were assessed in accordance with the guidelines [17]. Although the gold standard to diagnose PH is mean pulmonary artery pressure (PAP) by invasive right heart catheterization and, non-invasively, it should be echocardiographically defined by right ventricular systolic pressure (RVSP), the sum of TRPG and estimated right atrial pressure [18], data of inferior vena cava (IVC) was not available in the present registry. Therefore, the present study defined PH, elevated pulmonary artery pressure, as TRPG 30 mmHg or higher [16,19,20]. Mitral valve inflow velocities were measured from the apical 2-chamber or 4-chamber view (E-wave and A-wave). Similarly, Doppler from the mitral annulus (e’) was obtained from the four-chamber view at the lateral wall or septal wall. E/A and E/e’ ratios were calculated by dividing the E-wave velocity by the A-wave and e’, respectively [21].

### 2.3. Participants, Outcome Measures, and Follow-Up Period of the Study

After excluding patients whose TRPG data were unavailable (*n* = 216), the present study enrolled LAPLACE TAVI registry (*n* = 2056) patients who underwent TAVI between 17 May 2010 and 30 June 2021. Based on TRPG before TAVI, all participants were first divided into two groups, those without PH (TRPG < 30 mmHg): the “PH (−) group” (*n* = 1407, 61.9%), and those with PH as the “PH (+) group” (TRPG ≥ 30 mmHg) (*n* = 649, 28.6%). Next, based on the TRPG level shortly after TAVI (at 4.1 ± 5.3 days interval after the TAVI procedure), the PH (+) group was further subdivided into 2 groups; the “Recovered PH group” (TRPG after TAVI < 30 mmHg, *n* = 253) and the “Persistent PH group” (TRPG after TAVI ≥ 30 mmHg, *n* = 396) (Supplementary Figure S1).

The primary outcome measure was the composite of subsequent cardiovascular-related (CV) death and hospitalization due to heart failure that occurred following TAVI. Secondary endpoints were components of the primary endpoint: subsequent CV death and heart failure hospitalization, respectively. The longest follow-up for the primary outcome was 10.6 years, with median follow up of 1.8 years.

### 2.4. Statistical Analysis

Quantitative variables are presented as the mean ± standard deviation or median with interquartile range (IQR) in accordance with the results of the Shapiro-Wilk normality test. Categorical variables are presented as the number and percentage. Quantitative data across groups were compared using the ANOVA test or the Kruskal-Wallis test. Unadjusted Kaplan-Meier estimates evaluated the time to the cumulative incidence of endpoints followed by the log-rank test for comparisons. Univariate and multivariate analyses of Cox proportional hazards regression analyses calculated hazard ratios (HRs) of the TRPG as a continuous variable for endpoints. Variables used in multivariate models were selected based on background demographics and univariate analyses ( Supplementary Table S1). Consequently, in addition to the TRPG as a continuous variable, models used in Cox proportional hazard analyses for evaluating the risk of primary and secondary outcome measures included the following covariates: Model 1: TRPG (−10 mmHg) [22], age and male sex, and Model 2: TRPG (−10 mmHg), age, male, AF/AFL, peripheral arterial disease, pacemaker implantation, history of stroke, diabetes mellitus, hemoglobin, albumin, eGFR and Log NT-proBNP. Statistical significance was defined as a *p* value < 0.05 and analyses were performed using statistical software (JMP Pro 12.0; SAS Institute Inc, Cary, NC, USA and SPSS ver. 27; IBM Corp, Armonk, NY, USA).

## 3. Results

### 3.1. Baseline Demographics, Medications, Procedural Characteristics and Devices of PH (−) and Recovered and Persistent PH Groups within PH (+) Group

First, the characteristics of the background demographics, comorbidities, medications, procedures and devices were compared between the PH (−) and PH (+) groups (Table 1). Compared to the PH (−) group (*n* = 1407), patients in the PH (+) group (*n* = 649) were older, more likely to be men, to have history and worse of heart failure, more severe heart failure (according to NYHA heart failure classification), atrial fibrillation/atrial flutter (AF/AFL), history of coronary revascularization, and pacemaker implantation, while the incidences of conventional atherosclerotic risk factors, including hypertension, diabetes and dyslipidemia, were similar between the two groups. Procedural risk scores of TAVI were higher in the PH (+) group. Markers of cardiac and renal impairment and function, NT pro-BNP and serum creatinine, were significantly higher in the PH (+) group. The degrees of mitral and tricuspid regurgitation (MR and TR) revealed by echocardiography were significantly higher in patients in the PH (+) group. With respect to medications, the prevalence of taking beta-blockers, diuretics and anticoagulants were higher in the PH (+) group. Parameters regarding TAVI procedures and devices, such as approaches and type/size of transcatheter heart valve (THV) (balloon- vs. self-expandable), were similar in both groups. Thereafter, comparisons of the two groups within the PH (+) group, Recovered PH vs. Persistent PH were additionally performed (Table 2). There were no significant differences in age, BMI, NYHA heart failure classification, procedural risk scores, or most comorbidities other than AF/AFL between the Persistent and Recovered PH groups. Echocardiographic parameters related to AS, aortic valve area, transaortic valve pressure gradient (mean and max), and degrees of aortic and mitral regurgitation were not different between the two groups. However, left ventricular ejection fraction (LVEF) was paradoxically higher in the Persistent PH group. While there were no differences in the prevalence of taking beta-blockers, ACEI/ARB, statins and even diuretics, that of oral anticoagulants was higher in the Persistent PH group, probably due to the higher incidence of AF/AFL. Moreover, despite similar characteristics regarding the procedures and devices of TAVI, implanted THV size was significantly smaller in the Persistent PH group.

### 3.2. Long-Term Cumulative Incidences of the Composite of CV Death and Heart Failure Hospitalization Following TAVI in PH (−), Persistent PH and Recovered PH Groups

During the follow-up period, the primary outcome measure, the composite of CV death and heart failure hospitalization, occurred in 245 out of the 2056 participants (11.9%) and the incidences in each group were 10.0% (*n* = 141) in the PH (−) group, 11.9% (*n* = 30) in the Recovered PH group, and 18.7% (*n* = 74) in the Persistent PH group. The incidences of CV death among the entire subject population, and in the PH (−), Recovered and Persistent PH groups were 5.5% (*n* = 113), 4.3% (*n* = 61), 5.9% (*n* = 15) and 9.3% (*n* = 37), respectively. Similarly, the incidences of heart failure hospitalization were 8.1% (*n* = 166), 7.0% (*n* = 98), 6.3% (*n* = 16) and 13.1% (*n* = 52), respectively.

**Figure 1:**
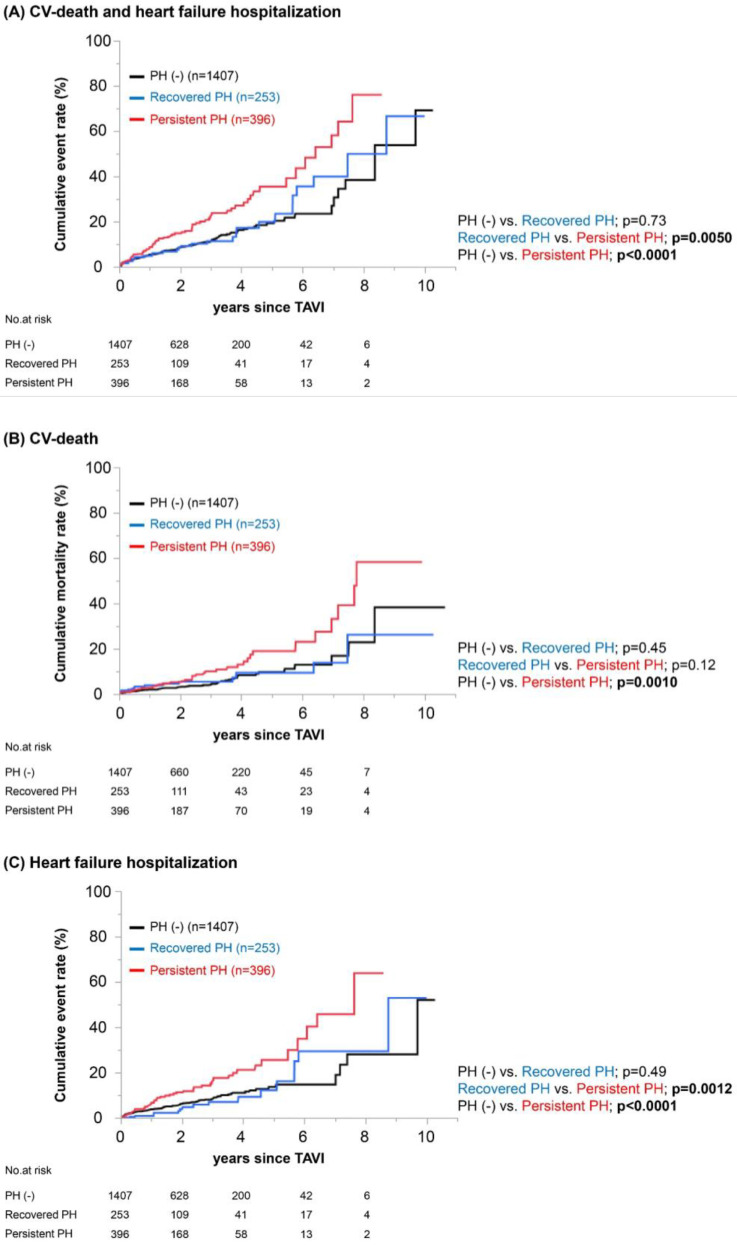
**Long-term outcomes following TAVI in the PH (−), Persistent PH and Recovered PH groups.** Figure legend: Kaplan-Meier estimates of patients in PH (−) (black), Recovered PH (blue) and Persistent PH (red) groups and survival tables indicating cumulative incidences of composite CV-death and heart failure hospitalization (**A**), CV-death (**B**) and heart failure hospitalization (**C**).

Unadjusted Kaplan-Meier analyses followed by log-rank comparisons showed a significantly higher cumulative incidence of the primary outcome measure in the Persistent PH group compared to the PH (−) and Recovered PH groups, while those in the PH (−) and Recovered PH groups were similar (Figure 1A). Kaplan-Meier estimates of CV death and heart failure hospitalization also demonstrated higher cumulative incidences in the Persistent PH group compared to those in the other 2 groups, which were similar (Figure 1B,C).

### 3.3. Unadjusted and Adjusted Cox Proportional Hazard Analyses Assessed the Prognostic Impact of the Reduction in TRPG through TAVI

**Figure 2:**
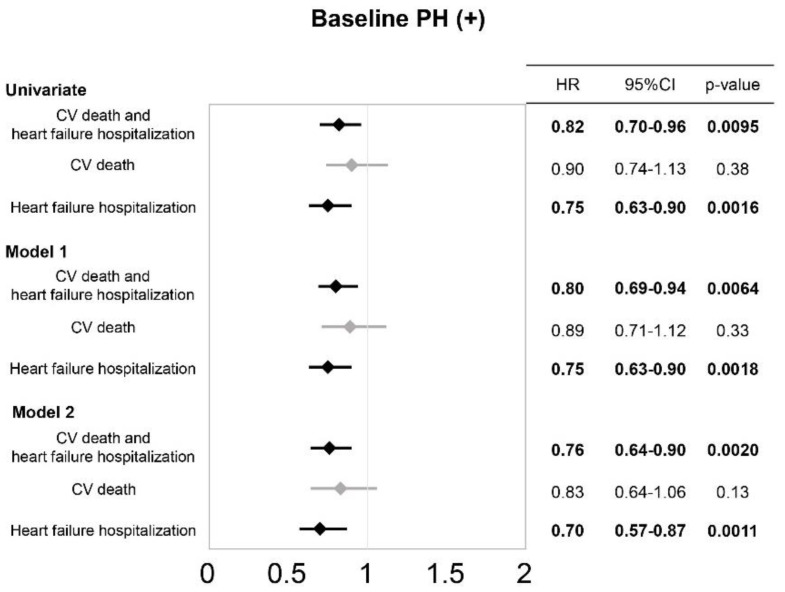
**Hazard ratios of TRPG in Baseline PH (+) derived from univariate and multivariable Cox proportional hazard analysis.** Hazard ratios of 10 mmHg decrease in TRPG (10 mmHg) derived from univariate and multivariable Cox proportional hazard analyses of primary endpoint, subsequent CV death and heart failure hospitalization in baseline PH (+) participants. Model 1 was adjusted by age, sex and TRPG. Model 2 was adjusted by age, sex, TRPG, AF/AFL, peripheral arterial disease, pacemaker implantation, history of stroke, diabetes mellitus, hemoglobin, albumin, eGFR, and Log NT-proBNP.

Univariate and multivariate Cox proportional hazard analyses evaluated the impact of the change in TRPG, as a continuous variable on the risk of primary and secondary outcome measures among all participants and those with PH before TAVI. In the multivariate analyses, a 10 mmHg decrease in TRPG was adjusted by variables which were identified in the univariate analyses (Supplementary Table S1), and those in comparisons of the background demographics between the Recovered and Persistent PH groups (Table 1). Univariate Cox proportional analyses identified diabetes, AF/AFL, history of stroke, peripheral artery disease (PAD), history of PMI, serum hemoglobin, serum albumin, estimated glomerular filtration rate (eGFR), and serum level of NT-proBNP to be significantly associated with risk of the primary endpoint. Accordingly, in addition to the unadjusted univariate analyses and an age- and sex-adjusted model (Model 1), TRPG decrease for the risk of primary and secondary endpoints were assessed by using Model 2 which included age, sex, diabetes, AF/AFL, history of PMI, history of stroke, serum hemoglobin, serum albumin, eGFR, and logarithm (log-) NT-proBNP. Consequently, in patients with PH before TAVI (PH (+)), 10 mmHg decrease in TRPG (−10 mmHg) was significantly associated with a decreased risk of the composite of CV death and heart failure hospitalization in both univariate and multivariate models (Figure 2) (HR: 0.82, 95% CI: 0.70–0.96, *p* = 0.0095, HR: 0.80, 95% CI: 0.69–0.94, *p* = 0.0064 and HR: 0.76, 95% CI: 0.64–0.90, *p* = 0.0020, respectively). Moreover, it was also an independent predictor for reduced risk of heart failure hospitalization in all analyses (HR: 0.75, 95% CI: 0.63–0.90, *p* = 0.0016, HR: 0.75, 95% CI: 0.63–0.90, *p* = 0.0018 and HR: 0.70, 95% CI: 0.57–0.87, *p* = 0.0011, respectively). Conversely, no significant association was found between TRPG decrease (−10 mmHg) and CV death in any models. Moreover, the same analyses in all participants in this study (both patients with PH (−) and (+) before TAVI) demonstrated that a decreased TRPG (−10 mmHg) was consistently associated with a reduced risk of heart failure hospitalization in univariate and multivariate analyses using both Model 1 and Model 2. Additionally, Cox analysis using Model 2 showed a significant association between decreased TRPG and lower risk of the primary outcome measure (Supplementary Figure S2).

### 3.4. Changes in Echocardiographic Indicators of Left Atrial Overload after TAVI in Recovered and Persistent PH Groups

**Figure 3:**
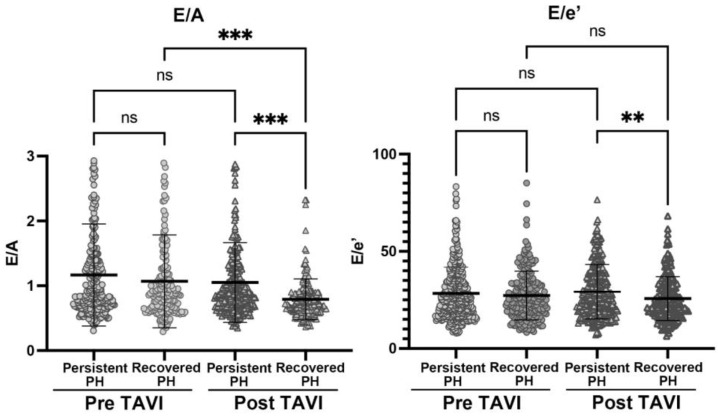
**Changes in E/A and E/e’ ratios through TAVI in patients with PH.** Figure legend: Pre- and post-TAVI E/A (**left**) and E/e’ (**right**) ratios in Persistent and Recovered PH groups. Com parisons using one way ANOVA calculated the significance in each group and timing of the measurement. **: *p* < 0.01 and ***: *p* < 0.001.

To elucidate potential insights into the mechanisms of the association between favorable outcomes and reduced TRPG through TAVI in this study, we evaluated pre- and post- TAVI echocardiographic parameters, which estimate left ventricular filling pressure, ratios of early (E) to late (atrial, A) diastolic transmitral flow velocity (E/A) and E to early diastolic mitral annular tissue velocity (E/e’), in the Persistent and Recovered PH groups. As shown in Figure 3 (left), despite similar E/A ratios before TAVI in both groups, it was significantly reduced in the Recovered PH group, while there was no change in the Persistent PH group. Moreover, the post-TAVI E/A ratio was significantly lower in the Recovered PH group. Similarly, the post TAVI E/e’ ratio was significantly lower in patients in the Recovered PH group and was slightly lower compared to pre-TAVI E/e’ (Figure 3, right).

## 4. Discussion

The present study evaluated the impact of the change in PH status, which was represented by echocardiographically measured TRPG before and shortly after TAVI, on long-term outcomes following TAVI, the composite of CV death and heart failure hospitalization, and CV death and heart failure hospitalization individually. The primary findings are (1) a substantial prevalence (28.6%) of PH (TRPG > 30 mmHg) in AS patients undergoing TAVI and 38.9% of such patients with PH recovered to a degree similar to those without PH after TAVI, (2) patients with Recovered PH after TAVI had significantly lower risk of the composite of CV death and heart failure hospitalization, as well as CV death and heart failure hospitalization individually, and (3) major echocardiographic parameters estimating diastolic dysfunction and LV filling pressure, the E/A and E/e’ ratios, were lowered through TAVI in patients in the Recovered PH group, while no change was observed in the Persistent PH group.

Recently, TAVI has become a common therapeutic strategy for patients with severe AS and its indication has been rapidly expanding to include those with low-intermediate risk, along with substantial advancement of devices and procedures in TAVI overtime [3,4,5,6,23], although it was originally initiated for those inoperable for SAVR. Accordingly, the more precise risk prediction after TAVI has been gaining further importance to assess therapeutic strategies for severe AS [24,25].

Previous studies have showed that PH is one of the common complications in severe AS patients [26]. The prevalence of PH determined by invasive right heart catheterization or non-invasive echocardiography had been reported to range from 29% to 56% in patients with severe AS [9,27,28,29], which is consistent with the present study (28.6% in patients undergoing TAVI). PH in patients with AS occurs due to LV pressure overload, diastolic (with or without systolic) dysfunction, with subsequent increase in left atrial pressure [29,30,31,32]. Previous studies have indicated that preoperative PH increased mortality risk and decreased long-term survival in patients who underwent aortic valve replacement [17]. The treatment of aortic stenosis by aortic valve replacement (SAVR and TAVI) improves hemodynamics followed by reduction in the overload in the LV, LA, mitral valve and pulmonary vasculature [33], which may lead to the resolution of PH. Indeed, residual PH after TAVI was associated with decreased long-term survival. However, the plausibility of the PH recovery by any AVR has been reported to be dependent on the degrees/stages of the AS-induced cardiovascular remodeling [34]. Moreover, while most previous studies evaluating the impact of baseline or the change in PH on outcomes utilized data measured using an invasive pressure method [33,34], the prognostic implication of the change in non-invasive TRPG through aortic valve replacement in AS patients has been rarely evaluated. TRPG is a reliable noninvasive echocardiographic parameter representing the pulmonary artery systolic pressure [15] and pulmonary vascular resistance [35]. In this study, this non-invasive echocardiographic measurement may reflect the status of pre/post TAVI right ventricular overload. Amongst all the participants, TRPG was not significantly changed by TAVI. However, in patients with baseline PH, the TRPG after TAVI was significantly lowered. Moreover, echocardiographic parameters included in the definition of diastolic dysfunction [21], such as the ratios E/A (Early/Atrial wave velocity) and E/e’ (early mitral inflow/annulus velocity) were lowered by TAVI in patients in the Recovered PH group. In contrast, there was no significant change in these parameters in patients with Persistent PH. These findings suggest possible potential relationships between TRPG and E/A or E/e’, in AS and post-TAVI patients, and partly indicate that TAVI may be effective not only in the recovery of PH, but also in reverse cardiac remodeling in those without PH after the point of no-return and may improve the prognosis. On the contrary, AS patients with excessively advanced cardiac remodeling may experience a limited beneficial effect of TAVI with respect to reduced risks of mortality and heart failure hospitalization [36]. The present findings that a reduction in TRPG in patients with PH through TAVI was significantly associated with decreased risks of subsequent heart failure hospitalization and CV death, may suggest the enhanced efficacy of an earlier indication of TAVI in AS patients prior to excessive CV remodeling manifesting PH. It is important to note that the impact of a 10 mmHg reduction in TRPG was associated with a decreased risk of the primary outcome in multivariate analysis and was predominantly driven by reduction in heart failure hospitalization.

This study should be interpreted in light of the following limitations. First, its retrospective nature with a relatively small sample size may have resulted in confounding factors that were unaccounted for and were not recorded or not even included in the multivariate models, despite the fact the TRPG was adjusted by various covariates in the multivariate analyses. Second, all analyses were based on TRPG, a non-invasive echocardiography-derived estimation, as a surrogate indicator of PH and systolic pulmonary arterial pressure and pulmonary vasculature resistance, which were not validated by invasive right heart catheterization [37]. Even with these limitations, the strength in this study utilizing data from a prospective multicenter registry of TAVI patients includes the detailed evaluations of the pre- and post-procedural echocardiographic data to predict long-term outcomes following TAVI, which may be informative in determining the indication and timing of TAVI in patients with severe AS.

## 5. Conclusions

This retrospective cohort analysis of a prospective TAVI registry database demonstrated a significant association between reduced TRPG after TAVI and decreased risk of the composite of CV death and heart failure hospitalization following TAVI in patients with PH compared to those with unchanged TRPG before and after TAVI.

## Figures and Tables

**Table 1:** Baseline characteristics of PH (+) vs. PH (−) groups.

	PH (−) *n* = 1407, 68.4%	PH (+) *n* = 649, 31.6%	*p*-Value
Age, years	84.1 ± 5.3	84.9 ± 5.3	0.0011
Male, *n*	494 (35.1%)	196 (30.2%)	0.0284
BMI ^1^, kg/m^2^	22.7 ± 3.7	21.8 ± 3.7	<0.0001
NYHA class III or IV, *n*	614 (44.1%)	371 (57.7%)	<0.0001
Logistic EuroSCORE, %	14.7 (14.2, 15.2)	18.6 (17.6, 19.7)	<0.0001
EuroSCOREII, %	5.8 (5.4, 6.1)	8.0 (7.3, 8.7)	<0.0001
STS-PROM, %	6.4 (6.2, 6.6)	8.3 (7.8, 8.7)	<0.0001
Comorbidities			
History of heart failure, *n*	221 (24.6%)	146 (40.0%)	<0.0001
Hypertension, *n*	1074 (76.7%)	499 (77.1%)	0.84
Diabetes mellitus, *n*	324 (23.0%)	140 (21.6%)	0.46
Dyslipidemia, *n*	797 (56.7%)	347 (53.5%)	0.18
AF/AFL ^2^, *n*	281 (20.2%)	216 (33.8%)	<0.0001
Cancer, *n*	270 (19.2%)	104 (16.0%)	0.08
History of stroke, *n*	166 (11.8%)	65 (10.0%)	0.23
COPD ^3^, *n*	119 (8.5%)	64 (10.0%)	0.29
CKD (stage 3 or more), *n*	933 (66.3%)	464 (71.5%)	0.0192
PAD ^4^, *n*	198 (14.1%)	129 (20.1%)	0.0007
OMI ^5^, *n*	72 (5.1%)	40 (6.2%)	0.33
History of coronary revascularization ^6^, *n*	332 (23.6%)	122 (18.8%)	0.0148
*p*-PTAV ^7^, *n*	30 (2.1%)	19 (2.9%)	0.27
PMI ^8^, *n*	72 (5.1%)	50 (5.9%)	0.0212
Laboratory data			
NT-proBNP ^9^, pg/mL	2704 (2139, 3270)	4777 (3710, 5844)	0.0002
Creatinine, mg/dL	0.98 ± 0.6	1.10 ± 0.9	0.0003
eGFR ^10^, ml/min	53.6 ± 18.4	50.3 ± 19.2	0.0002
Hemoglobin, g/dL	11.6 ± 1.6	11.3 ± 1.6	<0.0001
Albumin, g/dL	3.8 ± 0.4	3.7 ± 0.5	0.0001
Echocardiographic findings			
LVEF ^11^, %	61.1 ± 10.0	59.9 ± 12.4	0.0162
AVA, cm^2^	0.70 ± 0.21	0.66 ± 0.21	0.0002
Peak gradient, mmHg	84.8 ± 29.4	88.3 ± 33.8	0.0152
Mean gradient, mmHg	48.7 ± 17.9	51.0 ± 21.1	0.0128
AR ≥ moderate, *n*	113 (8.0%)	52 (8.0%)	0.99
MR ≥ moderate, *n*	76 (5.4%)	92 (14.2%)	<0.0001
TR ≥ moderate, *n*	33 (2.4%)	105 (16.2%)	<0.0001
TRPG, mmHg	21.9 ± 4.7	38.6 ± 9.4	<0.0001
Medications			
Beta-blockers	437 (31.1%)	281 (43.4%)	<0.0001
ACEIs ^12^/ARBs ^13^	747 (53.1%)	362 (55.9%)	0.24
Statins	741 (52.7%)	331 (51.1%)	0.50
Diuretics	580 (41.2%)	379 (58.5%)	<0.0001
Oral anticoagulants	311 (22.1%)	239 (36.8%)	<0.0001
Procedural variables			
Procedure time, min	82.5 (80.5, 85.0)	81.5 (78, 85)	0.64
Fluoroscopy time, min	22.3 (21.8, 22.9)	22.2 (21.3, 23.0)	0.73
Contrast medium volume, mL	62.8 (60.7, 65.0)	63.3 (60.0, 66.7)	0.80
Conscious sedation, *n*	954 (67.8%)	404 (62.3%)	0.0134
Transfemoral approach, *n*	1329 (94.9%)	599 (93.0%)	0.08
Valve size, mm	24.9 ± 2.4	24.7 ± 2.4	0.16
Balloon expandable, *n*	988 (71.3%)	413 (69.5%)	0.0096

^1^ body mass index, ^2^ atrial fibrillation or flutter, ^3^ chronic obstructive pulmonary disease, ^4^ peripheral artery disease, ^5^ old myocardial infarction, ^6^ status post percutaneous coronary intervention or coronary artery bypass graft, ^7^ percutaneous transcatheter aortic valvuloplasty, ^8^ pacemaker implantation, ^9^ N-terminal pro B-type natriuretic peptide, ^10^ estimated glomerular filtration rate, ^11^ left ventricular ejection fraction, ^12^ angiotensin II receptor blockers, ^13^ angiotensin II receptor blockers.

**Table 2:** Baseline characteristics of Recovered vs. Persistent PH groups.

	Recovered PH *n* = 253, 39.0%	Persistent PH *n* = 396, 61.0%	*p*-Value
Age, years	84.5 ± 5.1	85.1 ± 5.4	0.15
Male, *n*	89 (35.2%)	107 (27.0%)	0.0273
BMI, kg/m^2^	21.6 ± 3.5	21.9 ± 3.7	0.24
NYHA class III or IV, *n*	149 (59.4)	222 (56.6%)	0.49
Logistic EuroSCORE, %	18.8 (17.1, 20.6)	18.5 (17.3, 19.8)	0.78
EuroSCOREII, %	8.2 (7.0, 9.4)	7.9 (7.0, 8.7)	0.64
STS-PROM, %	8.1 (7.4, 8.8)	8.4 (7.8, 9.0)	0.57
Comorbidities			
History of heart failure, *n*	52 (35.9%)	94 (37.6%)	0.73
Hypertension, *n*	188 (74.6%)	311 (78.7%)	0.22
Diabetes mellitus, *n*	51 (20.2%)	89 (22.5%)	0.48
Dyslipidemia, *n*	128 (50.6%)	219 (55.3%)	0.24
AF/AFL, *n*	68 (27.4%)	148 (37.9%)	0.0066
Cancer, *n*	36 (14.2%)	68 (17.2%)	0.32
History of stroke, *n*	30 (11.9%)	35 (8.8%)	0.21
COPD, *n*	26 (10.4%)	38 (9.7%)	0.77
CKD (stage 3 or more), *n*	174 (68.8%)	290 (73.2%)	0.22
PAD, *n*	55 (22.3%)	74 (18.7%)	0.27
OMI, *n*	20 (7.9%)	20 (5.1%)	0.14
History of coronary revascularization *n*	50 (19.8%)	72 (18.2%)	0.61
p-PTAV, *n*	5 (2.0%)	14 (2.4%)	0.25
PMI, *n*	17 (6.7%)	33 (8.3%)	0.45
Laboratory data			
NT-proBNP, pg/mL	4406 (3332, 5481)	4991 (3422, 6560)	0.60
Creatinine, mg/dL	1.09 ± 0.9	1.11 ± 0.9	0.72
eGFR, ml/min	52.2 ± 20.0	49.0 ± 18.9	0.0371
Hemoglobin, g/dL	11.5 ± 1.7	11.2 ± 1.6	0.0188
Albumin, g/dL	3.7 ± 0.5	3.7 ± 0.5	0.89
Echocardiographic findings			
LVEF, %	58.4 ± 13.8	60.9 ± 11.3	0.0138
AVA, cm^2^	0.65 ± 0.23	0.67 ± 0.20	0.33
Peak gradient, mmHg	89.5 ± 33.8	87.7 ± 34.0	0.50
Mean gradient, mmHg	52.2 ± 20.5	50.1 ± 21.5	0.24
AR ≥ moderate, *n*	24 (9.5%)	28 (7.1%)	0.27
MR ≥ moderate, *n*	34 (13.4%)	58 (14.7%)	0.67
TR ≥ moderate, *n*	30 (11.9%)	75 (18.9%)	0.0169
TRPG, mmHg	36.9 ± 9.0	39.7 ± 9.4	0.0001
Medications			
Beta-blockers	105 (41.7%)	176 (44.4%)	0.49
ACEIs/ARBs	138 (54.8%)	224 (56.6%)	0.65
Statins	129 (51.2%)	202 (51.0%)	0.96
Diuretics	148 (58.7%)	231 (58.3%)	0.92
Oral anticoagulants	81 (32.0%)	158 (39.9%)	0.0423
Procedural variables			
Procedure time, min	79.8 (75, 84)	82.5 (78, 87)	0.44
Fluoroscopy time, min	22.6 (21.3, 23.8)	21.9 (20.8, 23.0)	0.42
Contrast medium volume, mL	61.6 (56.2, 67.1)	64.4 (60.1, 68.7)	0.42
Conscious sedation, *n*	143 (56.5%)	261 (65.9%)	0.0161
Transfemoral approach, *n*	233 (93.2%)	366 (92.9%)	0.88
Valve size, mm	25.1 ± 2.4	24.5 ± 2.4	0.0028
Balloon expandable, *n*	156 (63.2%)	257 (67.1%)	0.31

## Data Availability

The data that support the findings of this study are available from the corresponding author, upon reasonable request.

## Supplementary Materials

The following supporting information can be downloaded at: https://www.mdpi.com/article/10.3390/jcm12020729/s1, Figure S1: Consort diagram of the study; Figure S2: Hazard ratios of TRPG in all participants derived from univariate and multivariable Cox proportional hazard analyses; Table S1: Univariate Cox proportional hazard analyses for predictors of CV-death and heart failure hospitalization following TAVI.
