# Supplementary material for: Favorable Prognosis in Patients with Recovered Pulmonary Hypertension after TAVI: An Analysis of the LAPLACE-TAVI Registry
Source: J Clin Med. 2023 Jan 16;12(2):729. doi: 10.3390/jcm12020729 (PMC9866150; doi:10.3390/jcm12020729)
Supplement: Supplementary file 1 [file jcm-12-00729-s001.zip › jcm-2112679-supplementary.pdf]

Supplementary Table S1: Univariate Cox proportional hazard analyses for predictors of CV-death and heart failure hospitalization following TAVI

	HR	95% CI	p-value
Age (1 year older)	1.02	0.99-1.06	0.24
Male	1.19	0.79-1.80	0.40
BMI[1] (1kg/m^2 higher)	0.97	0.92-1.02	0.28
Hypertension	0.95	0.59-1.54	0.83
Diabetes mellitus	1.69	1.10-2.59	**0.0160**
Dyslipidemia	0.78	0.53-1.16	0.22
AF/AFL[2]	1.66	1.10-2.50	**0.0162**
History of coronary revascularization	1.07	0.66-1.73	0.77
History of cancer	1.54	0.98-2.44	0.06
History of stroke	2.31	1.37-3.91	**0.0017**
History of heart failure hospitalization	1.21	0.77-1.90	0.41
COPD[3]	1.11	0.59-2.07	0.75
PAD[4]	1.69	1.10-2.59	**0.0158**
OMI[5]	1.05	0.43-2.58	0.92
Pulmonary dysfunction	1.00	0.65-1.53	1.00
PTAV[6]	1.62	0.75-3.53	0.22
PMI[7]	1.99	1.13-3.50	**0.0168**
LVEF[8] (%) (1 SD higher)	0.94	0.77-1.15	0.52
AVA (cm^2) (1 SD higher)	1.01	0.81-1.25	0.93
Peak gradient (mmHg) (1 SD higher)	0.89	0.73-1.08	0.24
Mean gradient (mmHg) (1 SD higher)	0.88	0.71-1.08	0.23
TRPG (mmHg) (10 mmHg lower)	0.82	0.70-0.95	**0.0095**
Beta-blockers	1.12	0.76-1.66	0.56
ACEIs[9]/ARBs[10]	0.80	0.54-1.19	0.27
Statins	0.86	0.58-1.27	0.44
Diuretics	1.36	0.91-2.03	0.14

Oral anticoagulants	1.43	0.95-2.14	0.08
Hemoglobin (g/dL) (1 SD higher)	0.73	0.59-0.90	**0.0033**
eGFR[11] (mL/min/1.73m^2) (1 SD higher)	0.74	0.60-0.91	**0.0051**
Albumin (mg/dL) (1 SD higher)	0.75	0.61-0.93	**0.0071**
Log NT-proBNP[12] (pg/ml) (1 SD higher)	1.57	1.25-1.97	**0.0001**

1 body mass index, 2 atrial fibrillation or flutter 3 chronic obstructive pulmonary disease 4 peripheral artery disease 5 old myocardial infarction 6 percutaneous transcatheter aortic valvuloplasty 7 pacemaker implantation 8 left ventricular ejection fraction, 9 angiotensin converting enzyme inhibitors, 10 angiotensin II receptor blockers, 11 estimated glomerular filtration rate, 12 N-terminal pro B-type natriuretic peptide

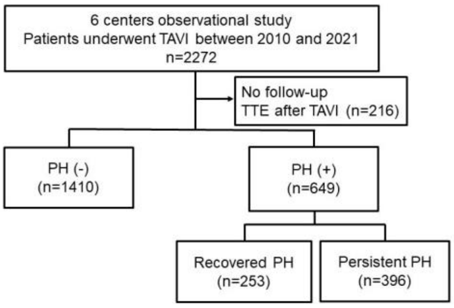

Figure S1: Consort diagram of the study

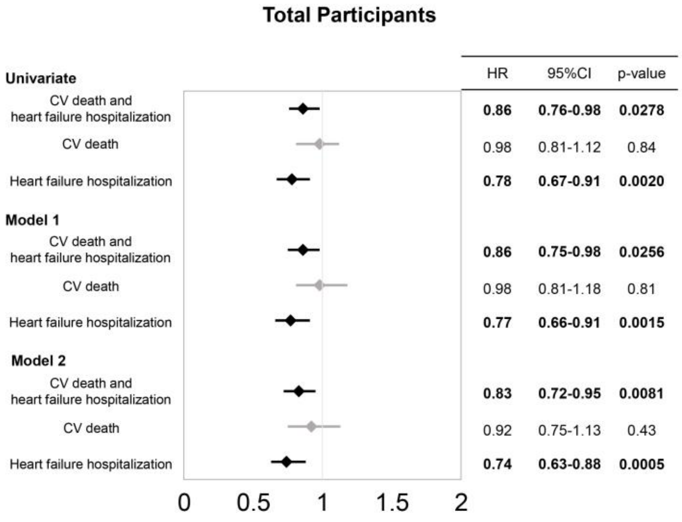

Figure S2: Hazard ratios of TRPG in all participants derived from univariate and multivariable Cox proportional hazard analyses
